# Main Poisonous and Allergenic Plant Species in Sicilian Gardens and Parks: Applications and Recommendations for Use

**DOI:** 10.3390/plants13071031

**Published:** 2024-04-05

**Authors:** Gianniantonio Domina, Emilio Di Gristina, Giulio Barone

**Affiliations:** Department of Agriculture, Food and Forest Sciences, University of Palermo, Viale delle Scienze Bldg. 4, 90128 Palermo, Italy; gianniantonio.domina@unipa.it (G.D.); giulio.barone01@unipa.it (G.B.)

**Keywords:** ecosystem services, human well-being, Italy, ornamental plants, pet well-being, public green, public health, toxic plants, urban biodiversity, weeds

## Abstract

This study identified the most common poisonous and allergenic plants occurring in Sicilian gardens and parks. Based on a survey conducted at 100 sites, a list was drawn up that reports the main biological and toxicological characteristics and ornamental uses of these plants. A total of 137 taxa were recorded, of which 108 were poisonous and 32 were allergenic. The most represented families were the Solanaceae, Moraceae, Apocynaceae and Fabaceae. The most represented geographical contingents were the European and the Mediterranean. A large number of toxic and allergenic plants recorded in Sicilian parks and gardens cause gastrointestinal disorders, 21 of which are deadly poisonous. Based on the results, actions for the management of existing gardens and the construction of new ones are discussed. The importance of environmental education for the population starting from school age is stressed. These recommendations aim to preserve cultivated biodiversity and, at the same time, protect human and pet health.

## 1. Introduction

Urban greenery constitutes an integral aspect of the ecosystem services provided by nature, encompassing processes such as water and air purification, and the provision of recreational spaces like urban parks [1]. However, the selection of plant species to be used has historically been approached predominantly from aesthetic or functional perspectives rather than considering their impact on human health. In planning and managing public and private green areas, the possible dangers of plants for human and companion animal health must be considered. The species to be used as ornamental greenery, in theory, should not have any harmful effects, but this is difficult to achieve. A walk in a southern Italian city is enough to realize how common toxic plants are and the risk to which the population is potentially exposed. The most striking case in Sicily is represented by oleander (*Nerium oleander* L.), a deadly poisonous plant [2] that is extensively used for ornamental greenery due to the beauty of its flowers and rusticity. Oleander and many other plants can cause gastrointestinal, cardiovascular, respiratory, neurological and immune system disorders and death in severe cases in humans and pets [3]. Most poisonous plants have a bad taste that discourages their consumption [2]; nevertheless, every year, unwary pickers and children must seek medical treatment following their consumption [4].

It is not always easy to define the concept of a poisonous plant and its danger because the effect on living organisms varies considerably depending on factors intrinsic to the plant and the organism with which it has come into contact [3].

Many plants that are cultivated by humans as ornamentals or that grow as weeds in green areas are used in traditional and modern pharmacopeias to treat various illnesses [5]. Even *Conium maculatum* L., commonly known as hemlock, a poisonous plant sadly known since ancient times, has been used as an analgesic and anti-inflammatory [6] and for the treatment of cervical carcinoma [7]. The dosage of the active compounds is one of the most random variables because it varies in the plant depending on the organ, season and environmental conditions [8]. Similarly, the presence of multiple bioactive compounds in plant organs can lead to combination interactions, synergy or additivity [9,10].

Even when plants are considered safe, they can also become toxic if they are grown in contact with organic or inorganic toxic substances present in the soil or air [11]. Indirect contact due to consumption by animals that have fed on poisonous plants or honey produced by bees that have foraged on poisonous species, such as *Rhododendron* sp. pl. or *Senecio* sp. pl., has been reported to be dangerous [12,13]. This generates a state of uncertainty regarding the consumption of plants and their derivatives that do not come from a controlled supply chain.

Frequently, media reports highlight incidents where unsuspecting foragers mistakenly gather poisonous plants, believing them to be edible. 

Even ornamental plants can be used for food purposes. It is a tradition in Sicily to collect and market the young shoots of *Ruscus hypophyllum* L. grown to make flowerbed borders. However, every day, the news reports cases of accidental ingestion of toxic plant parts by children, unwary people or pets [14]. From 1995 to 2007, 8564 cases of plant poisoning were reported by the Milan anti-poison unit [14]. The ingestion of *R. hypophyllum* and *R. aculeatus* L. berries has been the cause of several poisoning cases in Italy [4]. Cases of plant poisoning in Italy represent 1.3% of those found in hospital facilities [15]. For many geophytes, the most toxic parts are bulbs; the use of these species as ornamental essences would therefore seem safe, but in parks and gardens, children or digging animals can also come into contact with these underground organs [14,16].

Pollen allergies manifest in respiratory symptoms such as allergic rhinitis, conjunctivitis and bronchial asthma, exhibiting a distinct seasonality and recurring throughout the year due to the periodic release of various pollen types. In urban areas, the situation is further complicated by the combined impacts of climate change, including rising temperatures, increased humidity, extreme weather events and the urban heat island effect, alongside air pollution. The proliferation of allergenic pollens is also associated with specific plant species present in urban environments, as highlighted by Stach et al. [17], and the introduction of non-native, invasive and allergenic species such as *Ailanthus altissima* (Mill.) Swingle. In the management of green areas, plants responsible for respiratory allergies should not be overlooked; they can cause serious complications even without direct contact [18]. It is estimated that 10–30% of the world’s population suffers from pollen-related allergic rhinitis [19]. It is estimated that 50% of Europeans aged 15–64 suffer from at least one form of allergy, and the cost of asthma alone in Europe is around EUR 19.3 billion [19].

This field of investigation is little developed; studies have been carried out in Berlin and Munich, Germany [20]; Zagreb, Croatia [21]; Bursa, Turkey [22]; Latium, central Italy [23]; eastern Sicily [24]; and Milan, northern Italy [14]. An assessment of potential allergenic plants in the Royal Park of Portici in southern Italy was made [25]. The allergenic capacity of 150 of the most common urban trees in Mediterranean urban forests was evaluated [26]. A poster with a list of 265 plants of potential allergenic concern grown in Sicilian parks and gardens was presented at the 112° Congress of the Italian Botanical Society [27].

Sicily is the largest island in the Mediterranean Basin. It has a surface area of 25,832.39 km² and a population of 4,791,993 inhabitants living in 391 municipalities. The territory is 61.4% hilly, 24.4% mountainous and only 14.2% flat [28]. The highest peak is Etna, which exceeds 3350 m above sea level. The main island is surrounded by 15 smaller islands as well as a series of islets. Broadly speaking, according to [29], the predominate formations are carbonate rocks in western, northern and south-eastern Sicily, clayey–marly and evaporitic complexes in the central part, phyllite shale–crystalline rocks in north-eastern Sicily and volcanic substrates on Etna. A spectrum of 11 distinct bioclimatic zones spanning from the Upper Mediterranean to the Upper Cryomediterranean has been identified for Sicily [30]. Most of the population lives in urban centers located in flat areas. The great development of urban centers began in 1960 [31]. Thus, many peri-urban agricultural areas have been transformed into residential areas. The characteristics of green spaces differ depending on the history of each municipality, but shared characteristics can be outlined. In historic centers, there are usually few green spaces. Most of the historic greenery is concentrated in parks and villas that were established starting from 1700 on the edges of the inhabited centers that existed at the time. The residential development areas that grew up after the Second World War are characterized by new green spaces and wide, tree-lined avenues. Small municipalities, especially those close to large cities and their satellite towns, represent an ornamental and natural green basin of considerable importance, responding to the ever-increasing demand for naturalness on the part of the urban population. Historical ornamental parks are characterized by a large number of species, often represented by single individuals [32], while modern sites have a smaller number of species, of which many individuals are used. The main purpose of this contribution is to provide an analysis of the most common plants used as ornamentals in Sicily to understand the scale of the phenomenon and to provide a tool for planners and managers of public and private properties to be able to make informed choices of the plants to be used in new plantings and in the management of existing structures. This is the first comprehensive study with this aim performed in Sicily.

In detail, we inquired about (1) the identity, distribution and frequency of poisonous plants within Sicilian parks and gardens. (2) What is the occurrence rate of toxic wild plants in Sicilian parks and gardens? (3) Which highly toxic plants, presenting significant risks to humans and pets, occur in Sicilian parks and gardens, and what management approaches could mitigate these risks? Our aim was to evaluate the overall equilibrium between negative impacts (such as poisoning incidents in humans and pets) and positive contributions (including plant diversity, species preservation, and bird and pollinator sustenance) to ecosystems.

## 2. Results

**Figure 1:**
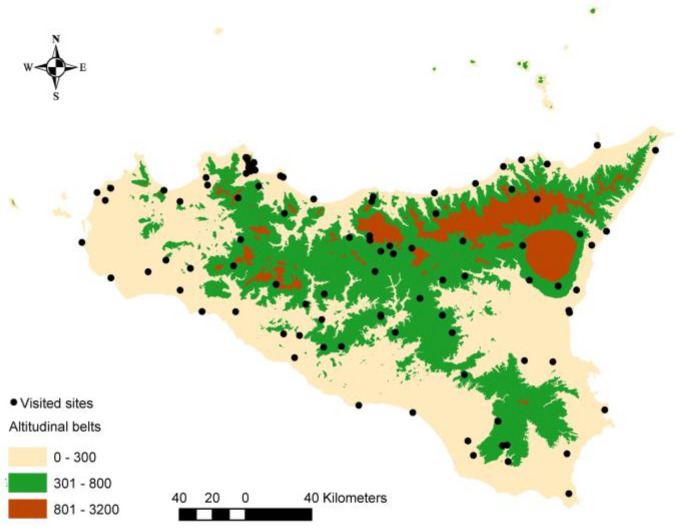
Distribution of studied sites.

Overall, 137 dangerous taxa were recorded among the plants most commonly found in 100 parks and gardens in Sicily (Figure 1, Table S1) because they were poisonous or highly allergenic. Concerning the frequency of the recorded taxa, 62 were very common, 58 were moderately common and 17 were less common.

Among the studied gardens, seven species stand out as particularly hazardous due to their deadly poisonous nature [33,34,35,36,37,38] and frequent occurrence. These species were *Drimia pancration* (Steinh.) J.C. Manning & Goldblatt, *Melia azedarach* L., *Nerium oleander* L., *Nicotiana glauca* Grahm, *Ricinus communis* L., *Solanum linnaeanum* Hepper & P.-M.L. Jaeger and *Thuja occidentalis* L. While *D. pancration*, *N. glauca*, *R. communis* and *S. linneanum* are often considered weeds, they are sometimes intentionally cultivated for ornamental purposes.

The most commonly cultivated allergenic plants with anemophilous pollination [39,40,41] included *Cupressus sempervirens* L., *Olea europaea* L., *Pinus halepensis* Mill, *P. pinea* L., *Populus nigra* L. and *Quercus ilex* L. Additionally, *Parietaria judaica* L., a common weed, is virtually ubiquitous across all surveyed sites.

**Figure 2:**
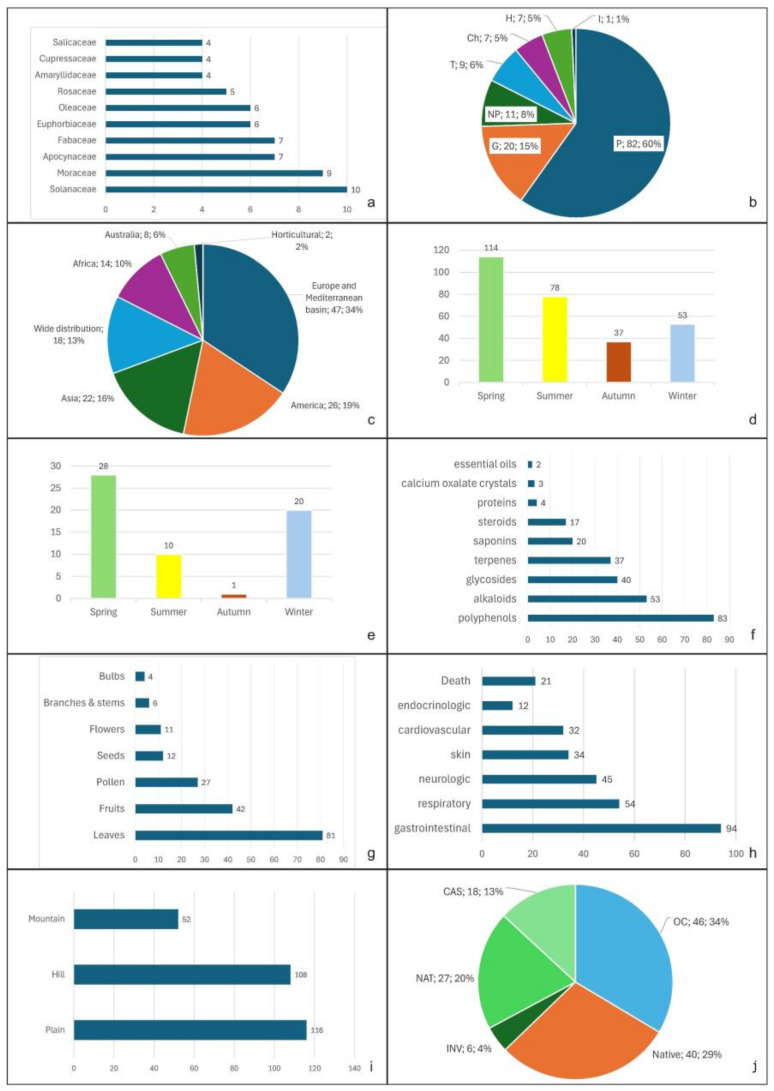
Characteristics of the poisonous and allergenic ornamental flora of Sicily: (**a**) the most represented families; (**b**) biological forms according to [42] (P, phanerophytes; NP, nanophanerophytes; Ch, chamaephytes; H, hemicryptophytes; G, geophytes; therophytes; I, idrophytes), number and percentage; (**c**) origins according to [43], number and percentage; (**d**) flowering seasons of the entire flora; (**e**) flowering seasons of the allergenic flora; (**f**) main classes of toxic principles according to [33,34,35,36]; (**g**) main parts causing health threats according to [14]; (**h**) potential disorders according to [33,34,35,36,37,38]; (**i**) altitudinal belts recorded; (**j**) status in Sicily (OC, only cultivated taxa; Native; CAS, casual aliens; NAT, naturalized aliens; INV, invasive aliens), number and percentage.

In the surveyed flora, the most represented families were Solanaceae (ten taxa), Moraceae (nine), Apocynaceae, Fabaceae (seven), Euphorbiaceae, Oleaceae (six), Rosaceae (five), Amaryllidaceae, Cupressaceae (four), Araceae, Asteraceae, Malvaceae, and Pinaceae (three), followed by another 49 families represented by one or two taxa (Figure 2a).

As biological forms [42], phanerophytes (82 taxa) and geophytes (20) dominated; nanophanerophytes (11), chamaephytes (7), hemicryptophytes (7) and hydrophytes (1) were less represented (Figure 2b). This spectrum respects that of historic Sicilian gardens [32]. This is due to the fact that trees, bushes and geophytes require less care than other plants. Annuals, in particular, mainly grow in private gardens, where the care and dedication of the owners are greater. Likewise, aquatic plants such as *Nymphaea alba* L. are underrepresented, primarily due to the scarcity of available water sources and the significant expense associated with their maintenance.

Regarding geographical origin [43], the most represented contingent was the European and Mediterranean (47 taxa), followed by American (27), Asian (22), widely distributed taxa (18), African (14), Australian (8) and taxa of horticultural origin (2) (Figure 2c). In historic Sicilian gardens [30], the American taxa were dominant, and the European and Mediterranean taxa only reached 20% of the total. Most recorded taxa were exotic (94), and 40 were native. 

Overall, 108 poisonous and 32 allergenic taxa were recorded. Of the poisonous taxa according to our classification based on the effects reported in the literature [33,34,35,36,37,38], 19 were highly toxic, 66 were toxic and 23 were moderately toxic.

In most cases, the entire plant was toxic; some taxa had non-dangerous parts, such as the arils of *Taxus baccata* L. [44] and the young shoots of *Dioscorea communis* (L.) Caddick & Wilkin and *Ruscus* spp., which are commonly consumed [45].

Pollen was mainly responsible for respiratory allergies (26 taxa). Stinging hairs present inside the fruits of *Brachychiton* spp. and *Lagunaria patersonia* (Andrews) G. Don were dangerous for the mucous membranes of the eyes and mouth [46]. In the genera *Cascabela*, *Ficus* and *Plumeria*, the irritating part was the latex [47,48].

In Sicilian gardens, poisonous and allergenic species flowered throughout the year with dominance in the spring (114 taxa) and summer (78) (Figure 2d). The most irritating allergenic species flowered mainly in spring (28 taxa) and winter (20) (Figure 2e).

The main classes of toxic principles [33,34,35,36,37,38] occurring in plants in Sicilian parks and gardens were polyphenols (reported for 83 taxa), alkaloids (53 taxa), glycosides (40), terpenes (37), saponins (20), steroids (17), proteins (4), calcium oxalate crystals (3) and essential oils (2) (Figure 2f).

The main parts causing health threats [14] are leaves and fruits (Figure 2g).

The large number of toxic and allergenic plants recorded in Sicilian parks and gardens are potential causes of gastrointestinal disorders (94 taxa), respiratory disorders (54), neurologic disorders (45), skin irritations (34), cardiovascular disorders (32) and endocrinologic disorders (12), and 21 taxa are reported [33,34,35,36,37,38] as deadly poisonous (Figure 2h).

Most of the plants detected grew in coastal areas (116) and hilly areas (108), while 52 taxa grew in mountain areas (Figure 2i).

Trees were most often used as single plants or to form rows, and bushes were grown as borders. Annuals and bulbs were used in flowerbeds and pots.

Regarding the tendency towards naturalization of these plants in Sicily [49,50], 46 taxa were only cultivated, and 18 were casual; i.e., they show a tendency towards naturalization but do not constitute populations independent from the cultivated plants. Of the taxa, 27 were naturalized, i.e., tended to form stable populations, and 6 were invasive and could constitute a risk not only for public health but also for biodiversity by competing with native species for spaces and resources (Figure 2j). *Cascabela thevetia* (L.) Lippold and *Melia azedarach* L. are deadly poisonous plants that have become naturalized in Sicily. These plants, along with their seeds, are readily accessible through nurseries and shops. *M. azedarach* is frequently employed as a street tree due to its fast growth and hardiness. However, it is advisable to discourage its use in new gardens.

Regarding the ecosystem services offered by these plants (data observed), 14 taxa had fruit regularly consumed by wild birds (mainly *Moraceae* and *Rosaceae*). A total of 19 taxa were foraged by bees, and 8 taxa performed both functions. This highlights the utility of these plants and underscores the importance of carefully managing their use, rather than advocating for their indiscriminate removal.

Up to 25 taxa pose potential risks to dogs and cats [51,52,53,54,55]. The most significant danger to these animals arises when they are allowed to roam freely, particularly in settings such as dog parks and private gardens adjacent to residential homes.

## 3. Discussion and Recommendations

The ornamental flora of Sicily has been identified as abundant in toxic and allergenic species, posing a significant public health concern. In the pursuit of promoting urban biodiversity, measures should prioritize heightened awareness regarding the utilization of existing resources. Ornamental poisonous plants also play an important ecosystem role. In addition to the ornamental aspect, such as the purification of the air and containment of noise and heat, 41 taxa were visited by bees or produced fruit serving as nourishment for the wildlife found in inhabited centers. This means that not all poisonous or allergenic plants must be removed, but their use and management must be controlled [22]. Given the presence of 25 toxic weeds in gardens, their monitoring and control are recommended. This work does not list the numerous native Poaceae present as weeds in parks and gardens; however, mechanical control in the spring–summer period in uncultivated areas close to hospitals and nursing homes would be desirable to reduce the risk of pollinosis [21,56]. Similarly, in these areas, the use of ornamental species responsible for pollinosis (*Pinus* sp. pl.; *Cupressus* sp. pl.; *Populus* sp. pl., etc.) should be avoided, or for dioecious plants, female individuals could be preferred [22]. This is because allergy prevention strategies center on removing allergens from the environment, reducing frequent exposure to strong allergens, and individuals avoiding contact with substances to which they have developed allergies [21]. Plants with a dual purpose, providing both productivity and ornamental value, such as hazelnut trees (*Corylus avellana* L.) warrant special consideration. While removing existing plants may be deemed impractical, their use may be discouraged in the planning of green spaces surrounding sensitive locations such as hospitals and retirement homes.

Management recommendations can be tailored according to the specific attributes of the analyzed green systems. In historical gardens with a limited number of species, sometimes consisting of just one individual, the presence of toxic or allergenic taxa may have a minimal impact. Conversely, in newly established green spaces where numerous individuals of the same species are present, there is potential for significant public health concerns, which should be addressed directly during the design phase to prevent adverse outcomes.

In the design of new green areas, especially those intended for children, toxic plants must be excluded, especially those with colorful fruit that can attract the attention of children. In existing parks, direct contact between people and toxic plants should also be limited through barriers. Explanatory tables with figures should be displayed in parks for users, explaining the dangers of the plants grown there and the contact details of the nearest poison control centers. Providing information about the flowering periods of prevalent allergenic plants can enhance awareness among allergy sufferers regarding their presence and potential impact. If the implementation of these measures proves ineffective, garden managers may contemplate the possibility of removing highly poisonous plants from gardens. Deadly poisonous species, such as *Cascabela thevetia* (L.) Lippold or *Nerium oleander*, should be removed from school gardens. In gardens frequented by children, these species should be grown as trees (scapose phanerophytes), not as bushes (caespitose phanerophytes), to reduce the risk of picking the leaves or fruits [57]. Similar precautions should be taken in both dog parks and private gardens where animals more easily come into contact with poisonous plants.

If foraged by bees, *Rhododendron simsii* Planch., *Ricinus communis* L. and *Senecio angulatus* L.f. can make honey poisonous. It is not advisable to place beehives in areas where these plants are very numerous or to cultivate these plants where beehives already are.

Promoting awareness about the ecological, phytosanitary and economic significance of urban green areas is a shared responsibility among all institutional bodies at the national, regional and local levels. Consequently, national-level awareness campaigns should be conducted through major media, and more targeted initiatives should be implemented at the local level. Urban residents frequently lack familiarity with plants, particularly those that are beneficial or hazardous. Organizing environmental education sessions in schools could prove highly beneficial in equipping citizens with a deeper understanding of the diverse plant resources available as well as the potential dangers they pose [58]. Informational materials may advise on the safe handling of mildly and moderately toxic plants, along with details on frequently misidentified species [20].

Cultivated flora, faster than spontaneous flora, undergoes changes over time. Our observations revealed a notable surge in the presence of new exotic species. This uptick can be attributed to heightened commercial plant exchanges facilitated by local and international nurseries in recent times [31,59]. To prevent potential confusion, which could result in significant health risks with toxic plants, nurseries should consistently provide the scientific names of the plants they offer for sale. They should provide buyers with information regarding the potential health risks associated with the plants they intend to purchase and, where feasible, recommend alternative species that pose fewer hazards. Even in the choice of new plants to be proposed to the general public, their potential danger should be considered, limiting the introduction of new poisonous species not yet present in the territory.

The results of this investigation and the suggested actions can likewise be extended to other regions around the globe characterized by a Mediterranean climate. Given that most ornamental garden and indoor plants are distributed worldwide irrespective of their native regions, comparable scenarios may arise in other geographical areas. However, given the peculiarities of the ornamental flora of the individual regions, it is our intention to further broaden the field of investigation to other areas in Italy.

## 4. Materials and Methods

We conducted our research in 100 public and private parks and gardens throughout the Sicilian territory (Figure 1, Table S1). The selection of surveyed sites aimed to provide a representative overview of the Sicilian situation, encompassing 63 historical gardens and 37 recently established (post-1950) green spaces. In terms of altitude, 51 sites are situated in the coastal region, 40 in hilly areas and 9 in the mountain belt. Public properties were predominantly chosen for easy accessibility. Urban areas were categorized by population size, with 35 sites located in small villages with fewer than 10,000 inhabitants, 33 in small towns (ranging from 10,000 to 50,000 inhabitants) and 32 in larger cities (exceeding 50,000 inhabitants). Each site was visited twice in spring and autumn between 2021 and 2023 to assess plant diversity. The entire surface of the gardens was explored on foot. The station parameters (coordinates, altitude, period of realization of the garden) and the presence of plants reported in the literature as poisonous and highly allergenic were recorded.

A list of toxic and allergenic species commonly used in Sicily as ornamental greenery that cause health problems for the population was drawn up (Table S2). Species present with very few individuals, in fewer than five localities, sometimes only within botanical gardens, were deliberately excluded. In the case of congeneric species that have similar properties and cause the same problems (e.g., *Ficus* spp. and *Pinus* spp.), only the most commonly used species were indicated, avoiding the repetition of the same information on numerous lines. The same goes for plants, such as apple trees (*Malus domestica* (Suckow) Borkh.), whose seeds contain amygdalin but in concentrations so low that such large quantities would have to be ingested as to make the threat more hypothetical than real [60]. Weeds that create problems commonly found in Sicilian gardens have also been included. The ornamental flora of Sicily was retrieved from specially conducted field surveys and from the literature [32,59,61,62,63,64]. The plant species were identified with the help of various works; in particular, reference was made to the Floras [65,66,67,68,69,70,71]. In some cases, a comparison with the living collections of the Botanical Garden of Palermo and the specimens of the *Herbarium Mediterraneum Panormitanum* in Palermo was performed. The taxa considered poisonous or allergenic in Refs. [5,33,34,35,36,37,38] have been included in our list. We classified plants as allergenic (A) and poisonous (P). These were divided by us into three hazard categories according to the literature information [33,34,35,36,37,38,51,52,53,54,55,56,57,58]: P1, highly toxic—for plants that can cause severe poisoning and death; P2, toxic—for plants that can cause poisoning, but death is excluded; P3, moderately toxic—for plants that cause light symptoms of poisoning that resolve on their own without the need for any intervention. For each species, the following information was recorded: family (according to [43]); biological form observed in Sicily (according to [42]); origin (derived from [43]); hazard (derived from [33,34,35,36,37,38]); poisonous part of the plant (derived from [33,34,35,36,37,38]); the part that most often causes problems for humans or dogs and cats (derived from [33,34,35,36,37,38] and from direct observation); the main toxic principles and side effects (derived from [5,33,34,35,36,37,38]); the ornamental type of use of the taxa in Sicily; their frequency in Sicily; altitudinal belt in Sicily; flowering period in Sicily (detected in the field); alien/native status in Sicily (derived from [49,50]); whether the plants produce fruit used by birds as food (detected in the field); and whether the plant is visited by bees (detected in the field). The plants were divided into three frequency classes: (1) very common, for those taxa that were found in more than half of the sites visited; (2) moderately common, for those taxa that were found in at least one-third of the sites surveyed; (3) less common, for those taxa that were found in less than one-third of the sites surveyed. The altitudinal belts were divided as follows: plain (P), from or up to 300 m; hill (H), between 301 and 800 m; and mountain (M), above 801 m. The indication of whether a plant was dangerous for dogs and cats was derived from [16,51,52,53,54,55].

## Data Availability

The original contributions presented in the study are included in the Supplementary Materials; further inquiries can be directed to the corresponding author.

## Supplementary Materials

The following supporting information can be downloaded at: https://www.mdpi.com/article/10.3390/plants13071031/s1, Table S1: Main poisonous and allergenic plant species in Sicilian gardens and parks; Table S2: Main poisonous and allergenic plant species in Sicilian gardens and parks.

## References

[1] Martinico F., La Rosa D., Privitera R. (2014). Green oriented urban development for urban ecosystem services provision in a medium sized city in southern Italy. iForest.

[2] Bulgarelli G., Flamigni S. (2010). Le Piante Tossiche E Velenose.

[3] Luzzi P. (1992). Piante Ornamentali Velenose.

[4] Moro P.A., Assisi F., Cassetti F., Bissoli M., Borghini R., Davanzo F., Della Puppa T., Dimasi V., Ferruzzi M., Giarratana T. (2009). Toxicological Hazards of Natural Environments: Clinical Reports from Poison Control Centre of Milan. Urban For. Urban Green..

[5] Benigni R., Capra C., Cattorini P.E. (1962). Piante Medicinali: Chimica Farmacologica e Terapia.

[6] Al-Snafi A.E. (2016). Pharmacology and Toxicology of *Conium maculatum*—A Review. Pharm. Chem. J..

[7] Mondal J., Panigrahi A.K., Khuda-Bukhsh A.R. (2014). Anticancer Potential of *Conium maculatum* Extract against Cancer Cells In Vitro: Drug-DNA Interaction and Its Ability to Induce Apoptosis through ROS Generation. Pharmacogn. Mag..

[8] Ginsburg H., Deharo E. (2011). A Call for Using Natural Compounds in the Development of New Antimalarial Treatments—An Introduction. Malar. J..

[9] Vaou N., Stavropoulou E., Voidarou C., Tsakris Z., Rozos G., Tsigalou C., Bezirtzoglou E. (2022). Interactions between Medical Plant-Derived Bioactive Compounds: Focus on Antimicrobial Combination Effects. Antibiotics.

[10] Masanotti G.M., Abbafati E., Petrella E., Vinciguerra S., Stracci F. (2019). Intensive Tobacco Cultivations, a Possible Public Health Risk?. Environ. Sci. Pollut. Res..

[11] Onat F.Y., Yegen B.C., Lawrence R., Oktay A., Oklay S. (1991). Mad Honey Poisoning in Man and Rat. Rev. Environ. Health.

[12] Mazokopakis E.E., Karagiannis C.G. (2022). Coturnism as a Cause of Deadly Rhabdomyolysis in Biblical Times. Clin. Kidney J..

[13] Yan S., Wang K., Al Naggar Y., Vander Heyden Y., Zhao L., Wu L., Xue X. (2022). Natural Plant Toxins in Honey: An Ignored Threat to Human Health. J. Hazard. Mater..

[14] Banfi E., Colombo M.L., Davanzo F., Falciola C., Galasso G., Martino E., Perego S. (2012). Piante velenose della Flora italiana nell’esperienza del Centro Antiveleni di Milano. Natura.

[15] Botti P., Cipriani F., Dannaoui B., Bravi S., Missanelli A. (2006). Gruppo Epintox Intossicazioni acute e avvelenamenti nei Dipartimenti di Emergenza e Urgenza in Italia. Ann. dell’Ist. Super. Sanità.

[16] Milewski L.M., Khan S.A. (2006). An Overview of Potentially Life-Threatening Poisonous Plants in Dogs and Cats. J. Vet. Emerg. Crit. Care.

[17] Stach A., Emberlin J., Smith M., Adams-Groom B., Myszkowskaet D. (2008). Factors that determine the severity of *Betula* spp. pollen seasons in Poland (Poznań and Krakow) and the United Kingdom (Worcester and London). Int. J. Biometeorol..

[18] Frenguelli G., Passaleva A. (2003). La scelta delle piante destinate al verde ornamentale. Giorn It Allergol. Immunol. Clin..

[19] Lauriola P., Talluri M. (2022). Tutti allergici? Il ruolo dei medici sentinella per l’ambiente per la prevenzione delle malattie allergiche respiratorie. Cesalpino.

[20] Sebald V., Schmack J., Egerer M. (2023). Occurrence and Diversity of Poisonous Plants in Urban Community Gardens. Renew. Agric. Food Syst..

[21] Židovec V., Jarić J., Poje M., Dujmović Purgar D. (2023). Poisonous and Allergenic Plant Species in Kindergarten Gardens in Novi Zagreb City Districts. J. Cent. Eur. Agric..

[22] Akdeniz N.S., Zencirkiran M. (2024). An Evaluation of Toxic Properties of Woody Landscape Plants Used in Hospital Garden Design. HERD.

[23] Leporatti M.L., Guarrera P.M., De Giacomo M. (1996). Wild and ornamental toxic plants in Latium region (Central Italy). Fitoterapia.

[24] Aleo N., Amato F., Aleo M. (2011). Le piante tossiche della flora trapanese (Sicilia). Quad. Bot. Ambient. Appl..

[25] Rispo M., De Masi L., Calandrelli M.M. (2020). Assessment of Allergenic Potential in Urban Forests: A Case Study of the Royal Park of Portici in Southern Italy. iForest.

[26] Cariñanos P., Marinangeli F. (2021). An Updated Proposal of the Potential Allergenicity of 150 Ornamental Trees and Shrubs in Mediterranean Cities. Urban For. Urban Green..

[27] Ciccarello S., Mazzola P., Spadaro V. Allergens in the park and garden flora of Sicily. Proceedings of the 112° Congresso della Società Botanica Italiana IV International Plant Science Conference (IPSC).

[28] Istituto Nazionale di Statistica. https://www.istat.it.

[29] Basilone L. (2018). Lithostratigraphy of Sicily.

[30] Bazan G., Marino P., Guarino R., Domina G., Schicchi R. (2015). Bioclimatology and vegetation series in Sicily: A geostatistical approach. Ann. Bot. Fenn..

[31] Domina G., Di Gristina E., Scafidi F., Calvo R., Venturella G., Gargano M. (2019). The Urban Vascular Flora of Palermo (Sicily, Italy). Plant Biosyst..

[32] Bazan G., Geraci A., Raimondo F.M. (2005). La componente floristica dei giardini storici siciliani. Quad. Bot. Ambient. Appl..

[33] Gastaldo P. (1987). Compendio della Flora Officinale Italiana.

[34] Bruneton J. (1995). Pharmacognosy, Phytochemistry, Medicinal Plants.

[35] Galliano Raspino M. (1996). Repertorio Fitoterapico.

[36] Pedretti M. (1997). Chimica e Farmacologia delle Piante Medicinali.

[37] Poppenga R.H., Luch A. (2010). Poisonous Plants. Molecular, Clinical and Environmental Toxicology: Volume 2: Clinical Toxicology.

[38] Colombo M.L., Assisi F., Puppa T.D., Moro P., Bissoli M., Borghini R., Perego S., Galasso G., Davanzo F. (2010). Most Commonly Plant Exposures and Intoxications from Outdoor Toxic Plants. J. Pharm. Sci..

[39] D’Amato G., Spieksma F.T.M., Bonini S. (1991). Allergenic Pollen and Pollinosis in Europe.

[40] Feliziani V. (1986). Pollini di Interesse Allergologico: Guida al Loro Riconoscimento.

[41] Ciampolini F., Cresti M. (1981). Atlante dei Principali Pollini Allergenici Presenti in Italia.

[42] Raunkiaer C. (1934). The Life Form of Plants and Statistical Geography.

[43] POWO Plants of the World Online. Facilitated by the Royal Botanic Gardens, Kew..

[44] Dumitra¸s D.-A., Bunea A., Vodnar D.C., Hanganu D., Pall E., Cenariu M., Gal A.F., Andrei S. (2022). Phytochemical Characterizationof *Taxus baccata* L. Aril with Emphasis on Evaluation of the Antiproliferative and Pro-Apoptotic Activity of Rhodoxanthin. Antioxidants.

[45] Di Tizio A., Łuczaj T.J., Quave C.L., Redži´c S., Pieroni A. (2012). Traditional Food and Herbal Uses of Wild Plants in the Ancient South-Slavic Diaspora of Mundimitar/Montemitro (Southern Italy). J. Ethnobiol. Ethnomed..

[46] Southcott R.V., Haegi L.A.R. (1992). Plant hair dermatitis. Med. J. Aust..

[47] Rajhans S., Pandya H., Kumar S.P., Bhadresha K., Yadav D.K., Rawal R., Ansari H., Dave R., Sindhav G. (2023). Assessment of Cytotoxic Effects of Latex from *Cascabela thevetia* (L.) Lippold and *Plumeria alba* L. via In vitro and In silico Approaches. J. Nat. Remedies.

[48] Mohammad H., Alzweiri M. (2022). Phytochemistry and pharmacological activities of *Ficus carica* latex: A systematic review. J. Chin. Pharm. Sci..

[49] Galasso G., Conti F., Peruzzi L., Alessandrini A., Ardenghi N.M.G., Bacchetta G., Banfi E., Barberis L., Bernardo L., Bouvet D. (2024). A second update to the checklist of the vascular flora alien to Italy. Plant Biosyst..

[50] Bartolucci F., Peruzzi L., Galasso G., Alessandrini A., Ardenghi N.M.G., Bacchetta G., Banfi E., Barberis G., Bernardo L., Bouvet D. (2024). A second update to the checklist of the vascular flora native to Italy. Plant Biosyst..

[51] Ghisleni G., Caretto F. (1999). Alcune piante d’appartamento tossiche per il cane e il gatto. Veterinaria.

[52] Botha C.J., Penrith M.-L. (2009). Potential Plant Poisonings in Dogs and Cats in Southern Africa: Review Article. J. S. Afr. Vet. Assoc..

[53] Berny P., Caloni F., Croubels S., Sachana M., Vandenbroucke V., Davanzo F., Guitart R. (2010). Animal Poisoning in Europe. Part 2: Companion Animals. Vet. J..

[54] Caloni F., Cortinovis C., Rivolta M., Alonge S., Davanzo F. (2013). Plant Poisoning in Domestic Animals: Epidemiological Data from an Italian Survey (2000–2011). Vet. Rec..

[55] Anadón A., Martínez-Larrañaga M.R., Ares I., Martínez M.A., Gupta R.C. (2018). Chapter 62—Poisonous Plants of the Europe. Veterinary Toxicology.

[56] Devarinti S.R. (2015). Pollen Allergy: Common Weeds in Telangana and Their Management Measures. JBFBP.

[57] Leveau P. (2021). Intoxications des enfants par les plantes. J. Pédiatr. Puéric..

[58] Davanzo F., Miaglia S., Perego S., Assisi F., Bissoli M., Borghini R., Cassetti F., Puppa T.D., Dimasi V., Falciola C. (2011). Plant Poisoning: Increasing Relevance, a Problem of Public Health and Education. North-Western Italy, Piedmont Region. J. Pharm. Sci. Res..

[59] Di Gristina E., Domina G., Barone G. (2021). The Alien Vascular Flora of Stromboli and Vulcano (Aeolian Islands, Italy). Ital. Bot..

[60] Bolarinwa I.F., Orfila C., Morgan M.R. (2015). Determination of amygdalin in apple seeds, fresh apples and processed apple juices. Food Chem..

[61] Mazzola P., Di Martino C. (1996). La florula decorativa del promontorio di Monte Pellegrino (Palermo). Quad. Bot. Ambient. Appl..

[62] Rossini Oliva S., Raimondo F.M., Valdés B. (2003). The ornamental flora of Western Sicily. Bocconea.

[63] Domina G., Mazzola P. (2008). Flora ornamentale delle isole circumsiciliane. Quad. Bot. Ambient. Appl..

[64] Schicchi R., Speciale M. (2020). Alberi di Palermo, Guida al Riconoscimento.

[65] Traverso O. (1926). Botanica Orticola.

[66] Bailey L.H. (1963). The Standard Cyclopedia of Horticolture, 1–3.

[67] Cullen J., Knees S.G., Cubey H.S. (2011). The European Garden Flora, 2nd ed., 1–5.

[68] Pignatti S. (2017). Flora d’Italia, 2nd ed., 1.

[69] Pignatti S. (2017). Flora d’Italia, 2nd ed., 2.

[70] Pignatti S. (2018). Flora d’Italia, 2nd ed., 3.

[71] Pignatti S., Guarino R., La Rosa M. (2019). Flora d’Italia, 2nd ed., 4 & Flora Digitale.

